# Evaluating ChatGPT’s Utility in Biologic Therapy for Systemic Lupus Erythematosus: Comparative Study of ChatGPT and Google Web Search

**DOI:** 10.2196/76458

**Published:** 2025-08-28

**Authors:** Kai Li, Yunfei Peng, Luyi Li, Bo Liu, Zhijian Huang

**Affiliations:** 1 School of Journalism and Communication, Guangxi University, No.100, Daxue East Road, Nanning, 530004, China, 86 13367611322; 2 Department of Rheumatology and Immunology, The Second Affiliated Hospital of Guangxi Medical University, Nanning, China

**Keywords:** biologic therapy, SLE, ChatGPT, google web search, health information, systemic lupus erythematosus

## Abstract

**Background:**

Systemic lupus erythematosus (SLE) is a life-threatening, multisystem autoimmune disease. Biologic therapy is a promising treatment for SLE. However, public understanding of this therapy is still insufficient, and the quality of related information on the internet varies, which affects patients’ acceptance of this treatment. The effectiveness of artificial intelligence technologies, such as ChatGPT (OpenAI), in knowledge dissemination within the health care field has attracted significant attention. Research on ChatGPT’s utility in answering questions regarding biologic therapy for SLE could promote the dissemination of this treatment.

**Objective:**

This study aimed to evaluate ChatGPT’s utility as a tool for users to obtain health information about biologic therapy for SLE.

**Methods:**

This study extracted 20 common questions related to biologic therapy for SLE, their corresponding answers, and the sources of these answers from both Google Web Search and ChatGPT-4o (OpenAI). Then, based on Rothwell’s classification, the questions were categorized into 3 main types: fact, policy, and value. The sources of the answers were classified into 5 categories: commercial, academic, medical practice, government, and social media. The accuracy and completeness of the answers were assessed using Likert scales. The readability of the answers was evaluated using the Flesch Reading Ease and Flesch-Kincaid Grade Level (FKGL) scores.

**Results:**

The study found that, in terms of question types, ChatGPT-4o had the highest proportion of fact questions (10/20), followed by policy (7/20) and value (3/20). Google Web Search had the highest proportion of fact questions (12/20), followed by value (5/20) and policy (3/20). In terms of website sources, ChatGPT-4o’s answers were sourced from 48 sources, with the majority coming from academic sources (29/48). Google Web Search provided answers from 20 sources, with an even distribution across all 5 categories. For accuracy, ChatGPT-4o’s mean score of 5.83 (SD 0.49) was higher than that of Google Web Search (mean 4.75, SD 0.94), with a mean difference of 1.08 (95% CI 0.61‐1.54). For completeness, ChatGPT-4o’s mean score of 2.88 (SD 0.32) was higher than that of Google Web Search (mean 1.68, SD 0.69), with a mean difference of 1.2 (95% CI 0.96‐1.44). For readability, the Flesch Reading Ease and Flesch-Kincaid Grade Level scores for ChatGPT-4o and Google Web Search were 11.7 and 14.9, and 16.2 and 20, respectively, indicating that both texts were of high reading difficulty, requiring readers to have a college graduate–level reading proficiency. When asking ChatGPT to respond at a sixth-grade level, the readability of the answers significantly improved.

**Conclusion:**

ChatGPT’s answers are characterized by accuracy, rigor, comprehensiveness, and professional supporting materials, and demonstrate humanistic care. However, the readability of the provided text is low, requiring users to have a college education background. Given the study’s limitations in question scope, comparison dimensions, research perspectives, and language types, further in-depth comparative research is recommended.

## Introduction

ChatGPT (OpenAI) has gained significant attention for its robust capabilities in content generation since its launch. It can respond to user inquiries by providing relevant, accurate, and evidence-based information in a logically coherent and easily understandable manner [1]. ChatGPT-4o (OpenAI) and its subsequent versions have more natural human-computer interaction capabilities, enabling them to respond to user commands more efficiently [2]. Although ChatGPT was originally developed neither for the health care domain [3] nor explicitly for answering medical questions [4], its content generation potential in the health care field is particularly noteworthy. Studies have found that ChatGPT plays a positive role in helping users gain health knowledge and answering medical inquiries [5,6].

Ayik et al [7] and Javaid et al [8] have indicated that ChatGPT can assist users in answering common questions in the health care domain. Temsah et al [9] noted that ChatGPT offers high-quality health care information, thereby bridging the gap in health care access. According to Ruksakulpiwat et al [10], the medical information provided by ChatGPT can facilitate communication between patients and health care providers, enhance patients’ self-care abilities, and improve treatment outcomes. Ayoub et al [5] are optimistic about ChatGPT’s potential for knowledge acquisition in the health care domain, suggesting that in certain situations, ChatGPT could enhance or even replace the commonly used Google Web Search, becoming an important information source for patients.

Systemic lupus erythematosus (SLE) is a multisystem autoimmune disease with a prevalence that varies across populations, with a global average prevalence ranging from 12 to 39 per 100,000 individuals [11]. China ranks among the highest in the world in terms of the burden of SLE [12], with a prevalence ranging from 30.13 to 70.41 per 100,000 individuals. The disease is most commonly observed in women of reproductive age, particularly those between 20 and 40 years old [11]. SLE leads to severe organ complications and may even be life-threatening [13], requiring urgent medical attention. In recent years, with in-depth research on the pathogenesis of SLE, targeted therapy has been proposed and has gradually gained attention. Some new biologics have been developed and introduced into clinical use. According to the 2023 annual “Patient Chart Dynamix: SLE (US)” and “Patient Chart Dynamix: SLE (EU5)” published by Spherix Global Insights, an increasing number of patients with SLE in the United States and 5 European countries are receiving biologic therapy, with the proportion of patients receiving such treatment rising by 32% compared with 2022 [14]. Currently, belimumab and anifrolumab have been successively approved by the Food and Drug Administration for the treatment of SLE [15]. In China, belimumab, telitacicept, and rituximab are the 3 biologic agents currently used in clinical practice [16].

Although biologic agents play a crucial role in SLE treatment, Liu et al [17] found that many patients with SLE lack knowledge about treatment options and disease management. This not only significantly affects their attitudes, treatment practices, and willingness to choose biologic therapy but also hinders their active participation in disease management, ultimately impacting their prognosis [17]. A study by Waldron et al [18] found that when health care providers fail to provide clear and consistent information during diagnosis, patients with SLE experience distress and confusion. In such situations, patients with SLE are highly likely to use the internet to meet their information needs [19]. According to the data from the Pew Research Center, 77% of health information seekers reported that their search began with applications such as Google, Bing, or Yahoo [20]. Jacquemart and Zweigenbaum [21] found that Google is the most effective search engine for retrieving answers to clinical questions. Lopes and Ribeiro [22] also found that Google significantly outperforms other search engines in accessing health information. Nowadays, with the emergence of large language models (LLMs), such as ChatGPT, some users are shifting from traditional web searches to these models as a primary source of health care information [5,6,23]. Traditional search engines, such as Google Bard and Microsoft Copilot, have also integrated LLMs into their platforms [24]. Hopkins et al [25] believed that LLMs (eg, ChatGPT) will fundamentally transform the way patients seek health care information.

The readability of health care texts directly impacts how effectively readers acquire health information from them. According to the readability assessment standards set by the National Institutes of Health and the American Medical Association, presenting health information on websites at a sixth-grade reading level or lower may help improve patients’ understanding of their condition and the treatment plans recommended by health care providers [26]. A study by Daraz et al [27] in the United States and Canada found that the vast majority of health information on the internet has poor readability, making it unsuitable for the general public. Furthermore, it may lead to misinformation and have a detrimental effect on health. Massie et al [28] noted that the readability of the American Society of Metabolic Surgery’s Patient Information Publications is poor and does not meet the standard of reading levels at or below the sixth grade. However, a study by Moons and Van Bulck [29] found that ChatGPT is a valuable tool for improving the readability and comprehensibility of health information. It can generate health care information suitable for the general public and easy to understand, based on user instructions, thereby addressing the issue of poor readability in health information on the internet [29].

Previous studies have evaluated the effectiveness of ChatGPT-3.5 (OpenAI) in responding to SLE-related questions and found that, in response to certain questions, its answers were not comprehensive or accurate and were sometimes unrelated to the questions [30]. ChatGPT-4o is widely recognized as a relatively new artificial intelligence model with a high user adoption rate [31]. However, no studies have yet evaluated the effectiveness of the new version, ChatGPT-4o, in responding to common SLE-related questions. Biologic therapy is a relatively new and important treatment for SLE and is regarded with high expectations. However, public awareness of this therapeutic approach remains insufficient [17]. Furthermore, the quality of SLE-related information on the internet varies, with some content containing advertisements and misleading information. This significantly hinders patients’ acceptance of biologic therapy [32], ultimately causing a significant negative impact on the clinical outcomes of patients with SLE. Therefore, this study aims to replicate users’ search behavior on Google Web Search to evaluate the utility of ChatGPT-4o in responding to common questions about biologic therapy for SLE. Specifically, we compare the following three aspects: (1) the most common questions related to biologic therapy for SLE, categorized by question types and topics; (2) the answers to these questions; and (3) the sources of these answers.

## Methods

### Overview

This study extracted 20 common questions related to biologic therapy for SLE, their corresponding answers, and the sources of these answers from both Google Web Search and ChatGPT-4o [33]. First, this study used the “People also ask” function in Google Web Search by entering the search terms “biologic therapy” and “systemic lupus erythematosus.” We selected the 20 most common questions about biologic therapy for SLE and recorded their answers and sources. Second, we instructed ChatGPT-4o to execute the command “Do a Google search with the search term ‘biologic therapy,’ ‘systemic lupus erythematosus,’ and record 20 common questions related to the search term.” Similarly, we recorded the questions, their answers, and the sources of those answers. Third, the obtained questions and the sources of answers were classified. Fourth, the accuracy, completeness, and readability of the answers were evaluated. In total, 2 rheumatology physicians (BL and ZH) were involved throughout the process of question selection and answer evaluation. In the event of a disagreement, a third physician was included in the discussion to reach a consensus.

### Google Web Search Operation

On July 17, 2024, a new installation of the Google Chrome browser was performed, and all browsing history, cookies, site data, cached images, files, sponsored websites, and advertisements were cleared before initiating the search. The keywords “biologic therapy” and “systemic lupus erythematosus” were entered simultaneously, and questions were identified using the “People Also Ask” function in Google Web Search. Additional related questions were obtained by clicking on each question.

We conducted 10 rounds of searches. During the selection of relevant questions, we found that once the sample size exceeded 30, the system started generating irrelevant or duplicate questions. Therefore, to ensure a representative sample, we selected only the first 30 questions from each round, yielding a total of 300 questions. After merging similar questions and analyzing their frequencies, 2 professional physicians (BL and ZH) assigned importance levels to each question according to their clinical experience. Finally, 20 questions related to biologic therapy for SLE were selected, along with their corresponding answers and the website information sources.

### ChatGPT Operation Execution

The following command was entered into ChatGPT-4o: “Do a Google search with the search term ‘biologic therapy,’ ‘systemic lupus erythematosus,’ and record 30 common questions related to the search term.” Then, each question was individually entered into ChatGPT-4o, and the corresponding answers and their sources were recorded. We conducted 10 rounds of searches, selecting 30 questions per round, which resulted in a total of 300 questions. We then used the same selection process for questions with Google Web Search, ultimately extracting 20 questions related to biologic therapy for SLE, along with their corresponding answers and website information sources.

Notably, unlike Google Web Search, ChatGPT-4o provided 2 or more sources for its answers. We used a 2-step verification process to assess whether hallucinations exist in its responses: First, each link attached to the answers was manually accessed and verified to check whether the link was valid and if the page content was consistent with the information provided in the answer. If the link was invalid or the page content was irrelevant to the answer, it was considered a potential indicator of hallucination. Second, based on the accuracy ratings given by 2 professional physicians (BL and ZH; refer to Accuracy and Completeness Evaluation section), if the average score of any answer was below 3, it was considered a hallucination, indicating that the answer contained factual errors or that the main information could not be substantiated [34]. No hallucination was found in the links or answers provided by ChatGPT after the 2-step verification. After removing duplicate links, we compiled statistics on the final website information sources.

### Inclusion and Exclusion Criteria

This study invited 2 professional physicians to screen and extract questions that are highly relevant to the topic and of significant concern to patients based on their clinical experience. The selected questions and answers were required to be closely related to biologic therapy for SLE. The inclusion criteria involved any question related to the terms “biologic therapy,” “biological therapy,” “biologics therapy,” “biologic treatment,” “rituximab,” “belimumab,” “anifrolumab,” and “systemic lupus erythematosus,” “lupus,” or “SLE.” The exclusion criteria included duplicate questions and questions unrelated to biologic therapy for SLE (eg, “What is the enemy of lupus?”).

### Ethical Considerations

This study analyzes publicly available data from Google Web Search and ChatGPT-4o. No personal or identifiable patient data were used. According to the Declaration of Helsinki established by the World Medical Association [35], this study does not involve human participants or personal data. Therefore, it is exempt from institutional review board review.

### Comparison Analysis

The common questions related to biologic therapy for SLE in this study were categorized using a modification of the Rothwell system [36]. According to this system, questions can be classified into 3 main types (fact, policy, and value), as well as 10 subtypes, that are education, accessibility, timeline of effectiveness, technical details, cost, indications or management, risks or complications, future prospects and challenges, longevity, and evaluation of treatment (refer to Table 1 for details). The website information sources were categorized into 5 types—commercial, academic, medical practice, government, and social media [33,37] (refer to Table 2 for details). Furthermore, 2 rheumatology physicians (BL and ZH) conducted question classification. In the event of disagreements, a third physician was brought in to achieve a consensus through discussion.

**Table 1.** Question types categorized by Rothwell’s classification [36]. The questions are categorized into 3 main types and subsequent subtypes.

Categorization	Explanation
Fact	Asks whether something is true and to what extent, objective information
Education	Educational information about biologic therapies for SLE^a^, not fitting into other subcategories
Accessibility	Where and how patients can get biologic treatment for SLE
Timeline of effectiveness	Length of time for the biologic therapy to show effectiveness in SLE, rather than just recovery milestones
Technical details	Details of how biologic therapies work for SLE, including dosing, method of delivery, and so on
Cost	Cost of treatment or rehabilitation
Policy	Asks whether a specific course of action should be taken to solve a problem
Indications or management	Therapy indications and alternatives, timing of biologic therapy for SLE, and posttreatment monitoring and management
Risks and complications	Risks and complications before, during, or after biologic treatment, including the rehabilitation period for SLE
Future prospects and challenges	The progress and difficulty in the development of biologic therapy for SLE
Value	Asks for the evaluation of an idea, object, or event
Longevity	Longevity and sustained effect of biologic therapy for patients with SLE
Evaluation of treatment	Successfulness, seriousness, or invasiveness of biologic therapy for SLE

a SLE: systemic lupus erythematosus.

**Table 2.** Categorization of website information sources.

Categorization	Explanation
Commercial	Organizations that provide public health information, including medical device, manufacturing, or pharmaceutical companies and news outlets
Academic	Universities, academic medical centers, or academic societies
Medical practice	Local hospitals or medical groups without clear academic affiliation
Government	Websites maintained by a national government
Social media	Blog, internet forums, support groups, and nonmedical organizations designed for information and video sharing

### Accuracy and Completeness Evaluation

The accuracy and completeness of responses generated by Google Web Search and ChatGPT-4o were evaluated by 2 professional physicians based on the guidelines published by the American College of Rheumatology [38]. Accuracy was assessed using a 6-point Likert scale (with 1=completely incorrect, 2=more incorrect than correct, 3=approximately equal correct and incorrect, 4=more correct than incorrect, 5=nearly entirely correct, and 6=completely correct). Completeness was evaluated using a 3-point Likert scale: 1 indicated “incomplete” (the response addressed some aspects of the question but omitted important information), 2 indicated “adequate” (the response addressed all aspects of the question with sufficient information for completeness), and 3 indicated “comprehensive” (the response addressed all aspects and provided additional information or context beyond expectations) [39]. Statistical analysis was performed using IBM SPSS Statistics 27, using descriptive statistics (mean and SD) and the Cohen κ test to assess the interrater reliability.

### Readability Measurement

Readability is defined as the ease of understanding or comprehension attributed to the writing style. Although many different readability tests exist, their measurement basically relies on two elements: (1) vocabulary and (2) word or sentence complexity (ie, the number of characters per space, syllables per word, or words per sentence) [40]. This study used the assessment tool “Readable” to evaluate the readability of the texts generated by Google Web Search and ChatGPT-4o [41]. Readability was assessed using the Flesch Reading Ease (FRE) and Flesch-Kincaid Grade Level (FKGL), both of which are commonly used to evaluate the readability of health-related literature on the internet [27,28]. The FRE score ranges from 0 to 100, with lower scores indicating higher reading difficulty [42]. The FKGL ranges from 0 to 18, representing the number of years of education required to comprehend the text. A lower score indicates easier readability [43].

## Results

### Question Analysis

The classification results for the 20 most common questions obtained from Google Web Search and ChatGPT-4o are presented in Table 3. Regarding the distribution of main question types, questions categorized under fact accounted for 12 from Google Web Search and 10 from ChatGPT-4o, those under policy for 3 and 7, and those under value for 5 and 3, respectively. Fact-category questions accounted for the highest proportion in both sources.

**Table 3.** 20 most common questions for biologic therapy for systemic lupus erythematosus from Google Web Search and ChatGPT-4o. The main types and subtypes of questions, categorized by Rothwell’s classification [36], are shown in parentheses.

	20 most common questions from Google Web Search	20 most common questions from ChatGPT-4o
1	What is the safest biologic for lupus? (Value, evaluation of treatment)	What is biologic therapy for SLE^a^? (Fact, education)
2	What is the new biologic for lupus? (Fact, education)	How do biologic therapies work in treating SLE? (Fact, technical details)
3	Is rituximab good for lupus? (Value, evaluation of treatment)	What are the most common biologic therapies used for SLE? (Fact, education)
4	What is the new drug for lupus approved by the FDA^b^? (Fact, accessibility)	What are the potential side effects of biologic therapies for SLE? (Policy, risks, and complications)
5	Can biologics cause lupus? (Policy, risks, and complications)	Are there any new biologic therapies being developed for SLE? (Fact, future prospects, and challenges)
6	How long does it take rituximab to work for lupus? (Fact, timeline of Effectiveness)	How does biologic therapy compare to traditional treatments for SLE? (Value, evaluation of treatment)
7	How effective is rituximab for lupus?(Value, evaluation of treatment)	Can biologic therapies be used in combination with other SLE treatments? (Policy, indications, or management)
8	What is the biologic treatment for systemic lupus erythematosus? (Fact, education)	How is the effectiveness of biologic therapies in SLE measured in clinical trials? (Fact, technical details)
9	Is rituximab FDA approved for SLE? (Fact, accessibility)	What are the long-term outcomes for SLE patients on biologic therapies? (Value, longevity)
10	What is the new treatment for lupus in the UK? (Fact, accessibility)	What is the cost and insurance coverage situation for biologic therapies for SLE? (Fact, cost)
11	Can rituximab cure lupus? (Value, evaluation of treatment)	How long does it take to see improvements with biologic therapy in lupus? (Fact, timeline of effectiveness)
12	How does rituximab work for SLE? (Fact, technical details)	What are the criteria for a patient with SLE to be eligible for biologic therapy? (Policy, indications, or management)
13	Is rituximab used to treat SLE? (Policy, indications, or management)	How do biologic therapies impact the overall quality of life for SLE patients? (Value, evaluation of treatment)
14	How do biologics help lupus? (Fact, technical details)	How often are biologic therapies administered for SLE patients? (Fact, technical details)
15	What is the best infusion for lupus? (Value, evaluation of treatment)	What is the role of Benlysta (belimumab) in SLE treatment? (Fact, technical details)
16	What is the self-injection for lupus? (Fact, technical details)	Are there any dietary or lifestyle changes recommended for SLE patients on biologic therapies? (Policy, indications, or management)
17	How often is Rituxan given for lupus?(Fact, technical details)	What are the approved biologic therapies for SLE? (Fact, education)
18	What are the indications for rituximab in SLE? (Policy, indications or management)	How are patients monitored while on biologic treatments for SLE? (Policy, indications, or management)
19	When will anifrolumab be FDA approved?(Fact, accessibility)	Are there any specific biologic therapies recommended for lupus nephritis? (Policy, indications or management)
20	Can adalimumab cause lupus? (Fact, risks and complications)	Can biologic therapies be used during pregnancy in SLE patients? (Policy, indications or management)

a SLE: systemic lupus erythematosus.

b FDA: Food and Drug Administration.

The distribution of question subtypes is illustrated in Multimedia Appendix 1. Questions from Google Web Search fell into 7 subtypes, with the highest proportions in the evaluation of treatment (n=5), followed by accessibility (n=4) and technical details (n=4). Other subtypes included education (n=2), risks and complications (n=2), indications or management (n=2), and timeline of effectiveness (n=1). Questions generated by ChatGPT-4o covered 9 subtypes, with indications or management (n=6) having the highest proportion, followed by technical details (n=4), education (n=3), evaluation of treatment (n=2). The remaining subtypes, including timeline of effectiveness, cost, risks and complications, future prospects and challenges, and longevity, were each represented by 1 question.

### Answer Analysis

Multimedia Appendix 2 presents the responses generated by ChatGPT-4o and Google Web Search. In terms of accuracy, the accuracy scores indicate that ChatGPT-4o achieved a higher mean score (mean 5.83, SD 0.49) compared with Google Web Search responses (mean 4.75, SD 0.94), with a mean difference of 1.08 (95% CI 0.61‐1.54). The ratings provided by the 2 physicians demonstrated good interrater reliability, with Cohen κ values of 0.72 for ChatGPT-4o and 0.78 for Google Web Search.

For completeness, ChatGPT-4o’s mean score was 2.88 (SD 0.32), significantly higher than Google Web Search’s 1.68 (SD 0.69), with a mean difference of 1.2 (95% CI 0.96‐1.44). The ratings also showed strong interrater reliability, with Cohen κ values of 0.94 for ChatGPT-4o and 0.88 for Google Web Search.

In terms of readability, the FRE and FKGL scores for Google Web Search and ChatGPT-4o were 16.2 and 20, and 11.7 and 14.9, respectively (Table 4). These results indicate that both texts have a high level of reading difficulty, requiring readers to have a college graduate-level reading proficiency [41]. In terms of response length, answers from Google Web Search were more concise, while those from ChatGPT-4o were more detailed and comprehensive.

**Table 4.** Accuracy, completeness, and readability of Google Web Search and ChatGPT-4o answers on biologic therapy for systemic lupus erythematosus.

	Item		Google Web Search	ChatGPT-4o
1	Accuracy, mean (SD)		5.83 (0.49)	4.75 (0.94)
2	Completeness, mean (SD)		2.88 (0.32)	1.68 (0.69)
3	Readability	
	FKGL^a^		16.2	11.7
FRE^b^		20	17.3

a FKGL: Flesch-Kincaid Grade Level.

b FRE: Flesch Reading Ease.

Furthermore, 3 of the 20 questions obtained from each source were similar (Table 5), but their answers differed significantly. The answers provided by Google Web Search were not comprehensive enough, lacking explanations of drug mechanisms and missing information on new medications (eg, Saphnelo) and emerging therapies (eg, chimeric antigen receptor–T cell therapy).

**Table 5.** Similar questions on biologic therapy for systemic lupus erythematosus of Google web search and ChatGPT-4o.

Question	Google Web Search	ChatGPT-4o
1	What is the new biologic for lupus?	Are there any new biologic therapies being developed for SLE^a^?
2	What is the biologic treatment for systemic lupus erythematosus?	What is biologic therapy for SLE?
3	How do biologics help lupus?	How do biologic therapies work in treating SLE?

a SLE: systemic lupus erythematosus.

### Analysis of Website Information Source

The distribution of website information sources differed notably between Google Web Search and ChatGPT-4o (Multimedia Appendix 3). Google Web Search provided answers from 20 sources: 2 commercial, 4 academic, 5 medical practice, 4 government, and 5 social media. ChatGPT-4o’s answers were cited from 48 sources, including 9 commercial, 29 academic, 1 medical practice, 2 government, and 7 social media sources. Notably, ChatGPT-4o relied more heavily on academic sources, whereas the source types of Google Web Search were more evenly distributed.

## Discussion

### Principal Findings

This study aimed to evaluate the utility of ChatGPT in providing users with health information on biologic therapy for SLE. The key findings are described further.

#### Accuracy and Rigor of Questions and Answers Provided by ChatGPT

On the one hand, the study found that ChatGPT-4o provided answers with higher accuracy than Google Web Search, which is consistent with the findings of Cohen et al [44]. On the other hand, among the 20 questions provided by Google Web Search, 9 were related to rituximab treatment for SLE, covering topics such as its accessibility, technical details, indications, and treatment evaluation. In contrast, the questions provided by ChatGPT-4o did not focus on rituximab treatment for SLE. There are significant differences between the questions regarding biologic therapy for SLE, which are of common concern to users, presented by both platforms. This finding is consistent with the findings of Dubin et al [33]. The reasons could be that Google provides an effective marketing channel for some pharmaceutical and health websites, enabling them to gain more exposure through bidding. Sponsored links are prioritized and displayed at the top or in prominent positions on the search results page [45-47]. However, many of these links may not only lack accurate clinical evidence but may also contain concerning misinformation [48]. In addition, search engine optimization techniques, through the optimization of website elements and the building of high-quality interlinks, effectively enhance a website’s ranking and visibility in search engines, making it a key channel for increasing organic traffic [49]. However, this could also mean that high-quality, unoptimized medical content pages may be pushed down in rankings, replaced by lower-quality pages [50]. ChatGPT-4o did not generate questions related to rituximab treatment for SLE. This may be because although textbooks such as “Harrison’s Principles of Internal Medicine” [51] and clinical guidelines such as “American College of Rheumatology Guidelines for Screening” [38] recommend the use of rituximab for treating SLE and its clinical application is widespread, rituximab has never been officially approved for SLE treatment at the legal level, nor is SLE listed as an indication in the drug’s prescribing information. The “Usage Policies” published by OpenAI under “Building with the OpenAI API Platform” explicitly state that ChatGPT-4o must not provide tailored medical or health advice without review by a qualified professional and without disclosing the use of artificial intelligence assistance and its potential limitations, as such actions may significantly impair the safety, well-being, or rights of others [52].

#### Comprehensive and Professional Nature of the Supporting Materials for the Answers Provided by ChatGPT

The study found that ChatGPT-4o provided answers with higher completeness than Google Web Search and provided information drawn from professional information sources, such as peer-reviewed journals, open-access academic publications from IntechOpen, and clinical practice guidelines from Kidney Disease: Improving Global Outcomes. This finding is consistent with the findings of Oeding et al [53]. This may be because ChatGPT’s training data primarily consists of a large volume of journal articles, books, reports, and other resources related to biomedical and health sciences, sourced from databases such as PubMed [54-56]. When responding to user queries, ChatGPT can quickly retrieve and integrate relevant information from its knowledge base. Based on this, compared with the previous study by Chen et al [57], we believe that ChatGPT can provide the general public with more professional answers regarding biologic therapy for SLE and help users better filter out false and misleading information from the internet.

#### Aspects of Humanistic Care in ChatGPT’s Answers

Based on content evaluation by 2 physicians, the study found that in ChatGPT-4o’s responses to 20 questions about biologic therapy for SLE, 50% reflected aspects of humanistic care, such as medication risk warnings, expressions of concern for patients’ physical and mental well-being, reminders about lifestyle adjustments, and considerations of medication affordability. This finding aligns with the research of Sorin et al [58] and Howe et al [59]. When providing answers regarding drug dosage and patient self-management of diseases, ChatGPT-4o includes recommendations at the end to seek guidance from health care providers. This reflects the rigor with which ChatGPT-4o delivers information to users. It also highlights the advantage of ChatGPT-4o, based on natural language processing technology, in effectively simulating human language and interaction patterns [60,61]. Compared with information on websites, which may contain logical inconsistencies or unclear statements, the way in which ChatGPT presents information is likely more accessible and comprehensible to the public. This helps to reduce the likelihood of patients being misled by vague or exaggerated information on advertising websites [57].

#### Low Readability of ChatGPT’s Answers

The study found that the texts generated by ChatGPT-4o regarding biologic therapy for SLE follow a general-specific-general structure, which aligns with human reading habits. However, the content’s readability was low. Based on the FRE and FKGL [27,28], the content provided by ChatGPT-4o regarding biologic therapy for SLE required a reading level equivalent to that of a college graduate [62], which is consistent with the findings of Ghanem et al [41]. The FRE score of Google Web Search was higher than that of ChatGPT-4o, which is consistent with the findings of Shen et al [63]. Possible reasons are as follows. First, ChatGPT was initially designed for a reading level comparable to that of high school students and above [29]. Second, ChatGPT’s training data and cited sources include high-quality medical literature, ensuring the accuracy of the medical terminology used in its responses. However, the use of medical jargon may reduce the readability of the text [64]. Third, ChatGPT generates responses based on patterns learned from large-scale text data, which typically requires readers to have a reading proficiency above the sixth-grade level [29]. However, when given explicit instructions to respond at a sixth-grade reading level, ChatGPT’s terminology density decreases, resulting in a significant improvement in the text’s readability. Specifically, the Flesch Kincaid Grade Level score decreased from 11.7 to 10.5, while the FRE score increased substantially from 17.3 to 51.9, indicating a marked enhancement in readability. This finding is consistent with that of Shen et al [63] and Mohammadi et al [65]. This capability enables ChatGPT to provide responses tailored to different educational levels, which may have significant implications for improving health care information accessibility and reducing health care disparities [66].

### Implications

First, this study compares ChatGPT-4o and Google Web Search in providing information on biologic therapy for SLE, offering a reference for the future dissemination of SLE treatment knowledge based on LLMs.

Second, this study conducts an in-depth analysis of ChatGPT-4o’s utility in providing information on biologic therapy for SLE, deepening understanding in both academia and industry regarding the potential of LLMs in delivering medical information and treatment recommendations.

Third, this study provides a reference for how users can use LLMs to access information on biologic therapy for SLE. It also offers insights into the effective use of LLMs for obtaining medical information to meet health care information needs.

### Limitations and Future Prospects

A few limitations should be noted in this study.

First, this study primarily focuses on common questions regarding biologic therapy for SLE, whereas in practice, users have a much broader range of concerns. Future studies can expand the scope of evaluated questions to gain a deeper understanding of ChatGPT’s utility in providing information on biologic therapy for SLE.

Second, this study only compares the basic characteristics of questions provided by Google and ChatGPT, including question types, answer sources, the accuracy, completeness, and readability of answers. Future research could broaden the comparison dimensions by including more factors.

Third, this study lacks a comparative analysis of ChatGPT-4o’s response characteristics and utility across different language contexts. Future research could focus on comparative studies across multiple languages.

Fourth, this study focuses on general internet users and does not account for differences across user groups. Future research could investigate the applicability and effectiveness of ChatGPT in meeting the information needs of various user groups.

Fifth, this study primarily focuses on evaluating information from a physician’s perspective, without reflecting patient or user concerns and experiences. Future research could explore the effectiveness of ChatGPT in addressing the information needs of users or patients through surveys or interviews, providing a deeper understanding of how well ChatGPT-generated content meets these needs.

### Conclusion

By comparing the utility of ChatGPT-4o and Google Web Search in providing information on biologic therapy for SLE, this study finds that ChatGPT has the potential to be more effective in delivering accurate medical knowledge and supporting patient education. However, given the limitations of this study in terms of question scope, comparison dimensions, language types, and research perspectives, further in-depth comparative research is recommended.

## Supplementary material

10.2196/76458 Multimedia Appendix 1 Subtype distribution of 20 most common questions on biologic therapy for systemic lupus erythematosus from Google Web Search and ChatGPT-4o.

10.2196/76458 Multimedia Appendix 2 The responses of ChatGPT-4o and Google Web Search.

10.2196/76458 Multimedia Appendix 3 tableDistribution of website information sources for Google Web Search and ChatGPT-4o answers on biologic therapy for systemic lupus erythematosus.

## Abbreviations

LLM: large language model
SLE: systemic lupus erythematosus
FRE: Flesch Reading Ease
FKGL: Flesch-Kincaid Grade Level
