# Supplementary figures and images for: Evaluating ChatGPT’s Utility in Biologic Therapy for Systemic Lupus Erythematosus: Comparative Study of ChatGPT and Google Web Search
Source: JMIR Form Res. 2025 Aug 28;9:e76458. doi: 10.2196/76458 (PMC12394579; doi:10.2196/76458)

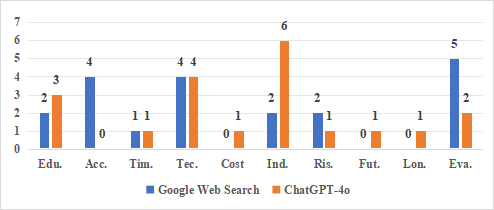

Supplement: Multimedia Appendix 1 [file formative-v9-e76458-s001.png]

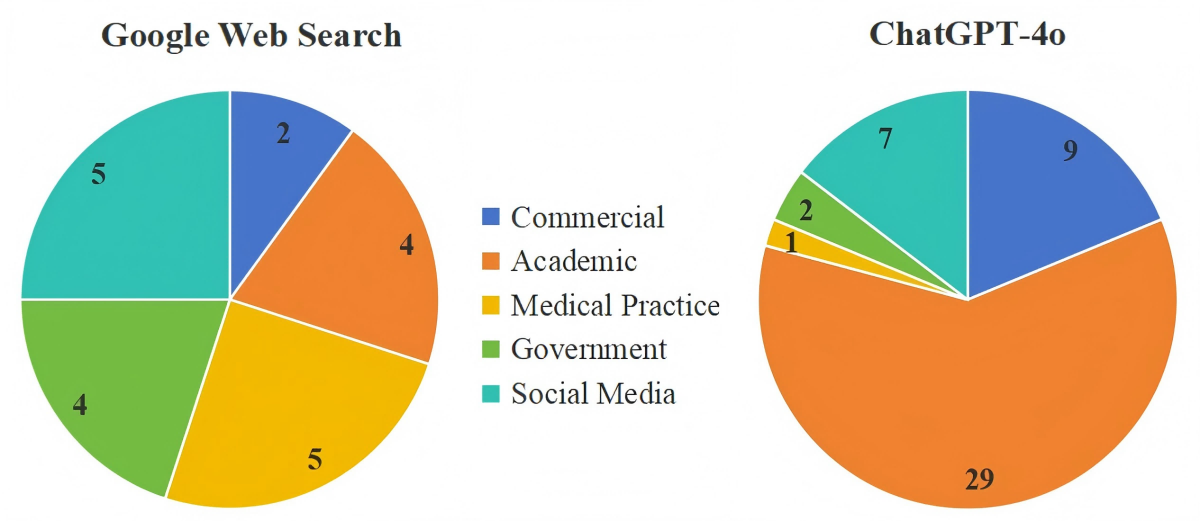

Supplement: Multimedia Appendix 3 [file formative-v9-e76458-s003.png]
